# Association Between Maternal Diet During Pregnancy and the Risk of Childhood Acute Lymphoblastic Leukemia. An Overview

**DOI:** 10.1002/cnr2.70231

**Published:** 2025-06-11

**Authors:** Kathya Torres‐Duarte, Laura Marcela Chávez Rodríguez, Catalina Mora‐Becerra, Jaime Moreno‐Chaparro, Hernando Gaitán‐Duarte

**Affiliations:** ^1^ Health Technologies and Policies Assessment Group (GETS), School of Medicine Universidad Nacional de Colombia Bogotá Colombia

**Keywords:** acute lymphoblastic leukemia, alcohol drinking, coffee, folic acid, maternal nutrition physiology

## Abstract

**Background:**

The effect of maternal habits during pregnancy has been studied to determine their association with the risk of childhood acute lymphoblastic leukemia (ALL).

**Aim:**

To synthesize current evidence on the association between maternal diet during pregnancy and the risk of childhood ALL.

**Methods:**

Overview of Systematic reviews (SRs) published until September 2023 were searched in MEDLINE (Pubmed), EMBASE, SCOPUS, Web of Science, and LILACS databases. Effect estimates and meta‐aggregate analysis results are presented according to maternal exposure subgroups: (I) alcohol; (II) animal protein; (III) coffee; (IV) fruits and vegetables consumption; and (V) folic acid supplementation. The risk of bias was assessed independently by two authors using the AMSTAR II tool. PROSPERO protocol CRD42023462391.

**Results:**

Thirteen SRs were included; of these, according to the AMSTAR II tool, three were graded as high‐quality, three as moderate‐quality, five as low‐quality, and two as critically low‐quality reviews. The inclusion of meat (fish), fruits, and vegetables in the diet during pregnancy, as well as folic acid supplementation, seems to have a protective effect against the development of childhood ALL. In contrast, the daily frequency and amount of coffee consumption during pregnancy could influence the development of this type of cancer in the pediatric population. We did not find enough evidence supporting the association of alcohol as a risk factor of childhood ALL.

**Conclusion:**

The low certainty of the evidence found makes it impossible to establish clear associations between maternal exposure during pregnancy to any of the five nutritional factors here evaluated and the development of childhood ALL.

## 1 Introduction

Acute lymphoblastic leukemia (ALL) is a cancer related to the malignant proliferation of lymphoid lineage cells in early stages of differentiation [1]. It is the most common cancer in the pediatric population aged 0–14 years (21% of cases) [2] and the second cause of mortality in this population group after accidents (e.g., traffic or drowning accidents, falls) [2, 3]. In the United States, the incidence of ALL in 2014 was 1.5 cases per 100 000 people, with an incidence peak in children aged 1–4 years and a progressive decline during adolescence and adulthood [1, 4]. In the case of Europe, the incidence of this type of cancer by 2012 was 6.7, 5.9, 5.3, and 4.8 cases per 10 000 children aged 0–14 years in Switzerland, Turkey, France, and England, respectively [5]. Furthermore, it has been reported that the incidence of ALL is 20% higher in Hispanic children and adolescents (respect to the white population) and that it affects slightly more boys than girls (2.8:2.2 ratio) [2, 6].

Since it is a blood cancer, ALL is characterized by rapid cancer cell invasion due to the mobilization of malignant blood cells through the bloodstream [7]. In addition, it has an increased growth and replication rate, typical of undifferentiated cells [3, 7]. These aberrations involve the mutation of a gene in a hematopoietic progenitor [8]. Currently, chromosomal abnormalities of prenatal and postnatal origin that intervene with tumor suppressor genes, oncogenes, and microRNA coding have been shown to be associated with ALL; however, they are only reported in 10%–30% of cases [1, 9, 10].

In view of the above, multiple external variables have been studied to find associations that contribute to the epigenetics of ALL [3]. As an example of the impact of environmental exposures, recent literature describes an association between most hematological malignancies and exposure to chemicals and ionizing radiation [10, 11, 12], due to their ability to accumulate in the bone marrow and subsequent accelerated metabolic activation in progenitor cell lines [7, 8, 9, 10, 12]. Equally, active and passive smoking has been implicated in the development of leukemia, mainly due to the genotoxic effects of benzene [10, 11, 13]. Exposure to household paints and its solvents has also been implicated as a risk factor for ALL by promoting primary DNA damage or secondary damage by activating disease subtypes resulting from chromosomal translocations in the postnatal period [14, 15].

Based on the above examples, and considering the described peak incidence of ALL in the pediatric population, it is reasonable to ask about the influence of maternal exposure during pregnancy and diet to certain external factors and the child's subsequent risk of developing the disease [11]. Therefore, in the present text, the information found has been divided into five main study groups (alcohol, coffee, fruit and vegetables, folic acid, and animal protein) in order to facilitate the collection, organization, and analysis of the information.

Regarding exposure to beverages, alcohol consumption has been shown to promote DNA methylation, a common marker of the development of leukemias and solid tumors [16]. Alcohol has also been implicated in the excessive production of inflammatory factors and free radicals, which may have long‐term effects on the quality of the genetic material of the mother and offspring [14]. Similarly, both tea and coffee intake have been proposed as risk factors of cancer as they contain topoisomerase II inhibitors, a fundamental enzyme in DNA synthesis and chromatin remodeling [3, 7, 11, 14].

On the other hand, it has been suggested that a balanced diet rich in proteins, fruits, and vegetables can act as a protective factor against the development of cancer [17], both in the general population and especially in pregnant women, and against the development of childhood blood cancers [18, 19, 20]. In addition, folic acid supplementation during pregnancy has also been reported to be a protective factor against the development of hematological neoplasms during childhood, especially ALL [21]. This would be based on the role of folate in DNA synthesis, repair, and methylation, which plays a crucial role against carcinogenesis. Similarly, vitamins C and D from fruit and vegetables prevent oxidative stress and act as coenzymes in many cell replication processes [21].

The design of the present study is of great importance for childhood ALL research, as the five exposure groups analyzed are modifiable factors related to lifestyle. Therefore, their early identification would play a fundamental role in primary prevention and in the development of future primary studies that will further investigate the interaction between genetics and environment in the process of childhood carcinogenesis [1, 7, 18]. By means of an overview study design, we aim to verify the quality of the available information, to synthesize and improve the accessibility of the current evidence, considering the controversy in the current literature regarding the association between maternal nutrition and the subsequent risk of developing ALL in children.

## 2 Methods

This study was designed as an overview (review of a systematic reviews). We followed the guidelines established in Chapter V (General Reviews of the Cochrane Handbook for Systematic Reviews of Interventions, version 6.4) [22], and used the Preferred Reporting Items for Systematic Reviews and Meta‐Analyses (PRISMA) [23]. In addition, the review protocol was registered in the Prospective International Registry of Systematic Reviews (PROSPERO) under code CRD42023462391 [24].

### 2.1 Selection Criteria

#### 2.1.1 Inclusion Criteria

- *Study types*: systematic reviews (SRs) with/without meta‐analysis.
- *Participants*: mothers of children diagnosed with ALL.
- *Types of exposure*: maternal exposure during pregnancy to the following factors: 1. alcohol; 2. animal protein; 3. coffee; 4. fruits and vegetables consumption; 5. folic acid supplementation.
- *Types of outcome*: measures of association between risk factors and the development of childhood ALL.

#### 2.1.2 Exclusion Criteria

- Studies involving mothers or children with other concomitant disorders were excluded.

### 2.2 Search Methods

An advanced search was conducted in: MEDLINE (Pubmed), EMBASE, SCOPUS, LILACS (Virtual Health Library), and Web of Science databases. Studies included until September 2023 were considered, and the search strategy included the use of controlled and free terms such as “leukemia,” “maternal,” “nutrition,” “dietary supplements,” “alcohol,” and “coffee.” Studies published in Spanish, English, French, and Portuguese were accepted. Both the search strategy and the search report are available in Supporting Information: Annex 1.

### 2.3 Additional Searches

We also conducted searches in sources such as Google Scholar and Open Grey (first five results pages). Additionally, a manual search was performed of the reference lists of the included reviews (snowball search method).

### 2.4 Data Selection and Extraction

The title and abstract screening stage was performed independently by two researchers. Then, using the Rayyan QCRI software [25], the screened studies were read in full text to decide on their final inclusion. Disagreements were resolved by a third reviewer. Then, data from the studies were independently extracted by one reviewer, and they were cross‐checked by another researcher. A preestablished and standardized format created in Microsoft Excel was used to organize the extracted data.

### 2.5 Quality Assessment

The selected studies were independently assessed using the AMSTAR II risk tool [26]. This tool is used to assess the methodological quality of SRs that include both randomized and non‐randomized studies. Its use in the evaluation of SRs includes the identification of research questions, search strategies, results, biases, and other statements necessary in a review [26, 27].

### 2.6 Data Synthesis

First, a descriptive analysis of the main characteristics of the SRs was made (authors, country of publication, participants, and setting). Then, the information was organized into the following exposure subgroups for analysis purposes: 1. alcohol; 2. animal protein; 3. coffee; 4. fruits and vegetables consumption; and 5. folic acid supplementation. Descriptive statistical analysis was performed by variable type (categorical or continuous), reporting relative and absolute frequencies (calculating totals, percentages, and ranges), as well as presenting measures of effect as reported in the included reviews.

The heterogeneity of the data reported in the SRs and differences in the methods used in the primary studies prevented us from doing a meta‐analytic synthesis of the evidence; therefore, findings are addressed by means of a meta‐aggregate description. In addition, we constructed a matrix to show the overlap of studies across SRs per exposure (see Supporting Information: Annex 2).

## 3 Results

### 3.1 Search Results

**FIGURE 1:**
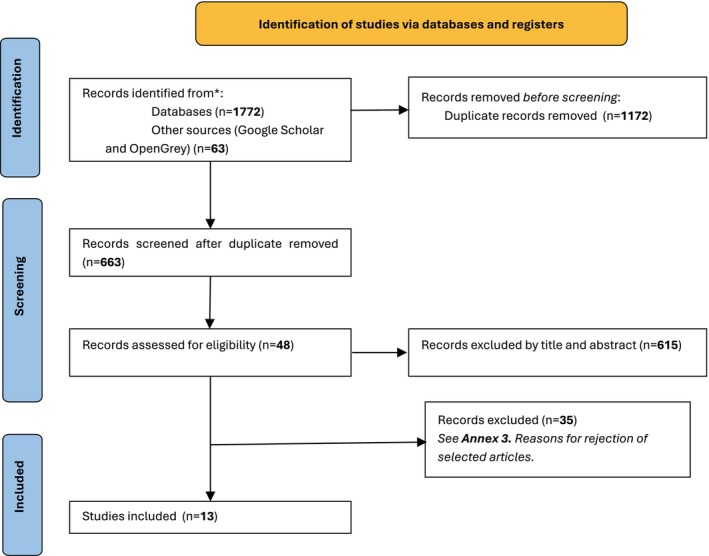
Flow diagram of the screening and selection process.

The searches identified a total of 1835 potentially relevant studies. After removing duplicates, 663 were screened by title and abstract reading, resulting in 48 records selected for full‐text reading to assess eligibility; of these, 35 did not meet the inclusion criteria: 27 were not SRs, five did not include a selected exposure group, two reported insufficient information, and one was written in a language not supported by the inclusion criteria (Supporting Information: Annex 3). Finally, 13 studies met the inclusion criteria and were included for data synthesis and analysis. The process of screening and inclusion is shown in Figure 1.

### 3.2 Description of the Studies

The 13 SRs included came from nine countries, being Greece (*n* = 3, 23.1%) and China (*n* = 3, 23.1%) the countries in which more studies were published. The remaining reviews came from Brazil (*n* = 1, 7.7%), Canada (*n* = 1, 7.7%), France (*n* = 1, 7.7%), Iran (*n* = 1, 7.7%), Malaysia (*n* = 1, 7.7%), several countries (France, Spain, Italy, Brazil, Belgium, USA, Canada, UK; *n* = 1, 7.7%), and the United States (*n* = 1, 7.7%). These studies were published between 1999 and 2023, and most of them between 2015 and 2017. The main characteristics of these 13 SRs are presented in Table 1.

**TABLE 1:** Characteristics of the studies included in the review.

References	Country	Population/participants	Number of included articles (*n*)	Included study designs	Exposure factors
Blot et al. [28]	United States	Children with cancer 645	13 (two leukemia related, but not ALL specifically)	Case–control	Animal protein
Infante‐Rivard et al. [29]	Canada	Children and adolescents with cancer 2385	33	Case–control	Alcohol
Latino‐Martel et al. [30]	France	Children and adolescents with cancer 5679	21 (13 with ALL discriminated)	Case–control	Alcohol
Zhang et al. [31]	China	Children with cancer 3815	10 (six ALL)	Case–control	Alcohol
Cheng et al. [32]	China	Children and adolescents with cancer 1686	7 (five ALL)	Case–control	Coffee
Brisson et al. [33]	Brazil	Children and adolescents with cancer 16 487^a^	Total 112 90 case–control pubs 22 reviews/meta‐analysis	Case–control Reviews/meta‐analysis	Alcohol
Thomopoulos et al. [34]	Greece	Children with cancer 2252	12 (11 maternal consumption)	Case–control or cohort	Coffee
Yan et al. [35]	China	Children with cancer 119 519^b^	49 (10 alcohol) (three coffee)	Case–control	Alcohol Coffee
Abiri et al. [36]	Iran	Children with cancer 9848	11	Case–control	Animal protein Alcohol Fruits and vegetables Folic acid
Dessypris et al. [37]	Greece	Children with cancer 8840^a^	18 (nine maternal diet, four maternal and childhood diet)	Case–control	Animal protein Fruits and vegetables Folic acid
Karalexi et al. [38]	Greece	Children with cancer 39 296	39 (20 ALL and maternal alcohol consumption, nine ALL and both maternal and paternal alcohol consumption)	Case–control	Alcohol
Ismail et al. [39]	Malaysia	Children and adolescents with cancer 6570	17 (11 discriminated ALL)	Case–control	Folic acid
Blanco‐Lopez et al. [40]	Multicountry	Children with cancer 28 292^a^	36	Cohort studies (one) Case–control studies	Animal protein Coffee Fruits and vegetables Folic acid

^a^ Participants with ALL were not separated from participants with other acute leukemias.

^b^ Without exposure grouping. It was not possible to determine the number of participants for chosen exposures in this study.

Overall, the population studied in the SRs was mothers of children and adolescents diagnosed with ALL (*n* = 11, 84.6%) [28, 29, 30, 31, 32, 34, 36, 37, 38, 39, 40] except for two: one in which the maximum age of the children and adolescents diagnosed with ALL was 21 years (*n* = 1, 7.7%) [33] and one in which all children were younger than 15 years (*n* = 1, 7.7%) [35]. These mothers were exposed to one or more of the above mentioned exposure subgroups.

Regarding the design of the primary studies included in the SRs, all included case–control and cohort studies. With respect to the number of primary studies included in the SRs, we found that two (15.4%) included between five and 10 studies [31, 32]; five (38.5%) included between 11 and 20 [28, 34, 36, 37, 39], and four included between 21 and 40 (*n* = 4, 30.8%) [29, 30, 38, 40]. The SRs by Yan et al. [35] and Brisson et al. [33] included 49 and 112 primary studies, respectively.

Regarding their distribution per exposure group, seven SRs (53.8%) addressed alcohol consumption [29, 30, 31, 33, 35, 36, 38]; four (30.8%) addressed animal protein consumption [28, 36, 37, 40]; four (30.8%) addressed coffee consumption [32, 34, 35, 40]; three (23.1%) addressed fruits and vegetables intake [36, 37, 40], and four (30.8%) addressed folic acid supplementation [36, 37, 39, 40].

Finally, in five SRs (38.5%), authors reported they had received funding [28, 29, 30, 35, 36] and only one (7.7%) informed of the existence of conflicts of interest [28].

### 3.3 Quality Assessment

According to the assessment of the 13 studies with the AMSTAR II tool, three (23.1%) were classified as high‐quality [35, 38, 40]; three (23.1%) as moderate‐quality [30, 34, 39]; five (38.5%) as low‐quality [31, 32, 33, 36, 37]; and two (15.4%) as critically low‐quality reviews [28, 29]. At a general level, all studies considered the risk of bias analysis and provided a satisfactory explanation for any heterogeneity observed. On the other hand, it was found that only one study reported on the sources of funding of the included studies; there were weaknesses in the searches, selection processes, and in the statistical pooling of results. The results reported by the two SRs classified as being critically low‐quality reviews [28, 29] were not included in order to obtain reliable results and maintain the quality of the present review. The results of the evaluation of the 13 SRs with the AMSTAR II tool are presented in Table 2.

**TABLE 2:** Evaluation of the included studies (AMSTAR II).

AMSTAR II																			
Author	1	2	3	4	5	6	7	8	9A	9B	10	11A	11B	12	13	14	15	16	Overall
Blot et al. [28]	Y	N	Y	N	N	N	N	Y	^a^	N	N	NM	NM	NM	Y	Y	NM	Y	Critically low
Infante‐Rivard et al. [29]	Y	N	Y	N	N	N	N	Y	^a^	N	N	NM	NM	NM	Y	Y	NM	Y	Critically low
Latino‐Martel et al. [30]	Y	Y	Y	PY	N	Y	PY	Y	^a^	PY	N	NM	Y	Y	Y	Y	Y	Y	Moderate
Zhang et al. [31]	Y	N	Y	PY	N	Y	PY	Y	^a^	PY	N	NM	NM	NM	Y	Y	NM	Y	Low
Cheng et al. [32]	Y	Y	Y	PY	Y	Y	Y	Y	^a^	Y	N	NM	Y	Y	Y	Y	Y	N	Moderate
Brisson et al. [33]	Y	PY	N	Y	Y	N	PY	Y	^a^	N	N	NM	NM	NM	Y	Y	NM	Y	Low
Thomopoulos et al. [34]	Y	Y	Y	PY	Y	Y	Y	Y	^a^	PY	N	NM	Y	Y	Y	Y	N	Y	Low
Yan et al. [35]	Y	Y	Y	PY	Y	Y	Y	PY	^a^	Y	N	NM	Y	Y	Y	Y	Y	Y	High
Abiri et al. [36]	Y	Y	Y	PY	Y	N	PY	Y	^a^	Y	N	NM	Y	Y	Y	Y	Y	Y	Moderate
Dessypris et al. [37]	Y	Y	Y	PY	Y	Y	PY	Y	^a^	Y	Y	NM	Y	Y	Y	Y	Y	Y	High
Karalexi et al. [38]	Y	PY	Y	PY	N	N	N	PY	^a^	PY	N	NM	Y	Y	Y	Y	Y	N	Low
Ismail et al. [39]	Y	PY	Y	PY	N	Y	PY	Y	^a^	N	N	NM	Y	N	Y	Y	Y	Y	Low
Blanco‐Lopez et al. [40]	Y	Y	Y	PY	Y	Y	PY	Y	^a^	PY	N	NM	Y	Y	Y	Y	Y	Y	High

*Note:* 1. Did the research questions and inclusion criteria for the review include the components of PICO? 2. Did the report of the review contain an explicit statement that the review methods were established prior to the conduct of the review and did the report justify any significant deviations from the protocol? 3. Did the review authors explain their selection of the study designs for inclusion in the review? 4. Did the review authors use a comprehensive literature search strategy? 5. Did the review authors perform study selection in duplicate? 6. Did the review authors perform data extraction in duplicate? 7. Did the review authors provide a list of excluded studies and justify the exclusions? 8. Did the review authors describe the included studies in adequate detail? 9A. Randomized controlled trials (RTCs)—Did the review authors use a satisfactory technique for assessing the risk of bias (RoB) in individual studies that were included in the review? 9B. Non‐randomized studies of interventions (NRSI)—Did the review authors use a satisfactory technique for assessing the risk of bias (RoB) in individual studies that were included in the review? 10. Did the review authors report on the sources of funding for the studies included in the review? 11A. RTCs—If meta‐analysis was performed, did the review authors use appropriate methods for statistical combination of results? 11B. NRSI—If meta‐analysis was performed, did the review authors use appropriate methods for statistical combination of results? 12. If meta‐analysis was performed, did the review authors assess the potential impact of RoB in individual studies on the results of the meta‐analysis or other evidence synthesis? 13. Did the review authors account for RoB in individual studies when interpreting/discussing the results of the review? 14. Did the review authors provide a satisfactory explanation for, and discussion of, any heterogeneity observed in the results of the review? 15. If they performed quantitative synthesis, did the review authors carry out an adequate investigation of publication bias (small study bias) and discuss its likely impact on the results of the review? 16. Did the review authors report any potential sources of conflict of interest, including any funding they received for conducting the review?

Abbreviations: N, no; NM, no meta‐analysis conducted; PY, partial yes; Y, yes.

^a^ Includes only NRSI.

### 3.4 Analysis by Maternal Exposure Groups

#### 3.4.1 Alcohol Consumption

Alcohol consumption was addressed in six SRs that included 86 primary studies, of which 21 were repeated across SRs (an overlap of 24.4%). The SR by Karalexi et al. [38] was considered the study that best addresses this maternal exposure subgroup since, out of the six SRs, it was the most recent one, the one with the highest quality, and the one in which most of the studies included in the other reviews were analyzed. According to the study by Karalexi et al. [38] as well as the other five SRs (see Table 3), there is not enough information supporting the association between alcohol during pregnancy and the development of childhood ALL. However, in their review, Yan et al. [35] reported that, after grouping the study population by regional subgroups, a discrete increased risk was found in North American mothers (OR 1.30; 95% CI 0.95–1.79) versus Australian mothers (OR 0.63; 95% CI 0.55–0.73) [35]. Similarly, after classifying the results according to the type of beverage, Latino‐Martel et al. [30] found an association between the consumption of liquors such as whiskey or vodka during pregnancy and the development of childhood ALL (OR 1.29; 95% CI 1.05–1.59).

**TABLE 3:** Reported associations between alcohol consumption during pregnancy and the development of childhood ALL.

References	Measures		Main results
Latino‐Martel et al. [30]	Pregnancy vs. no consumption	OR, 1.10; 95% CI (0.93–1.29)	No statistically significant association
	Pregnancy and ALL at age 0–4 years	OR, 1.17; 95% CI (0.83–1.65)	
	First trimester	OR, 0.91; 95% CI (0.67–1.25)	
	Second trimester	OR, 0.89; 95% CI (0.63–1.27)	
	Third trimester	OR, 0.82; 95% CI (0.62–1.10)	
	Spirits	OR, 1.29; 95% CI (1.05–1.59)	
Zhang et al. [31]	OR, 0.92; 95% CI (0.84–1.00)		Significant differences reported
Brisson et al. [33]	Third trimester	OR, 2.4; 95% Cl (1.1–5.4)	GSTM1‐null and CYP2E1*5 were associated with an increased risk especially for alcohol consumption during the third trimester of pregnancy
	Nursing period	OR, 4.9; 95% CI (1.4–16.6)	
Yan et al. [35]	Ever vs. never (total)	OR, 0.88; 95% CI (0.74–1.06)	No statistically significant association was observed
	Ever vs. never—Australian	OR, 0.63; 95% CI (0.55–0.73)	
	Ever vs. never—North American	OR, 1.30; 95% CI (0.95–1.79)	
	Ever vs. never—European	OR, 0.90; 95% CI (0.82–0.98)	
	Ever vs. never hospital‐based case–control	OR, 1.77; 95% CI (1.25–2.51)	
Abiri et al. [36]	Three studies showed no significant relationship^a^		Neutral effect of maternal alcohol consumption
Karalexi et al. [38]	Moderate consumption (< 2 glasses/week)	OR, 1.13; 95% CI (0.84–1.52)	No association between LLA and any level or type of alcohol consumption
	High consumption	OR, 0.98; 95% CI (0.71–1.36)	
	Any consumption	OR, 0.97; 95% CI (0.85–1.11)	
	Results were inconsistent		

^a^ This systematic review reports the measures of association that are informed by the primary studies; it does not provide a single (pooled) measure of association.

#### 3.4.2 Animal Protein Consumption

This maternal exposure subgroup was addressed by three SRs comprising 10 primary studies, five of which were repeated across the SRs (overlap of 50%). The SR that best approached this type of maternal exposure was the one conducted by Blanco‐López et al. [40], given that out of the three, this was the review most recently published and the one with the highest quality. According to the meta‐analysis performed by Blanco‐López et al., there is no association between maternal consumption of processed meats and the development of childhood ALL (OR 1.01; 95% CI 0.68–1.51) [40]. In addition, Dessypris et al., based on their meta‐analysis, reported that eating fish during pregnancy behaved as a protective factor against childhood ALL in children diagnosed before turning 5 years old (OR 0.27; 95% CI 0.14–0.53) [37]. This protective association is supported by the SRs conducted by Abiri et al. and Blanco‐López et al., as they also cite other studies with similar findings [36, 40]. In conclusion, the three SRs report results suggesting a protective effect of fish consumption during pregnancy against the development of childhood ALL [36, 37, 40] (see Table 4).

**TABLE 4:** Reported associations between animal protein consumption during pregnancy and the development of childhood ALL.

References	Measures	Main results
Abiri et al. [36]	Maternal consumption of beef may reduce the risk of childhood ALL^a^	Protective effects of maternal protein sources: beef and beans
	Fish consumption was related to lower ALL risk in some studies^a^	
Dessypris et al. [37]	Fish OR 0.27 (95% CI 0.14–0.53) (0–4 years old)	It was inversely associated with the risk of ALL (< 5 years)
	Association of cured and regular meats yielding null findings	
Blanco‐Lopez et al. [40]	Cured meats OR 1.01 (95% CI 0.68–1.51)	Maternal diet rich in protein sources could have a protective effect
	Statistically significant inverse association with beans and beef	

^a^ This systematic review reports the measures of association that are informed by the primary studies; it does not provide a single (pooled) measure of association.

#### 3.4.3 Coffee Intake

This type of maternal exposure was addressed by four SRs comprising 40 primary studies in total, with an overlap frequency of 22.5% (nine repeated studies across SRs). Three of these SRs report an association between the amount and frequency of coffee consumption during pregnancy and the risk of childhood ALL: Cheng et al. report a directly proportional association between maternal coffee consumption and childhood ALL since the probability of childhood ALL was higher in children of high‐frequency coffee drinkers during pregnancy (OR 1.65; 95% CI 1.28–2.12) compared to children of non/low‐frequency coffee drinkers (OR 1.26; 95% CI 1.05–1.50) [32]; Blanco‐Lopez et al. found that, compared to no consumption at all, consumption of more than two cups/day was associated with an increased risk (OR 1.45; 95% CI 1.12–1.89) [40], and Thomopoulos et al. found a positive association between high maternal consumption (three or more cups of coffee per day) and childhood ALL (OR 1.43; 95% CI 1.22–1.68) [34]. Only the SR by Yan et al. reports a weak and poorly significant association between this coffee consumption during pregnancy and childhood ALL [35]. (see Table 5).

**TABLE 5:** Reported associations between coffee consumption during pregnancy and the development of childhood ALL.

References	Measures		Main results
	Frequency/quantity	Odds ratio (OR)	
Cheng et al. [32]	Ever drinkers	OR 1.26 (95% CI 1.05–1.50)	Consumption: high vs. non‐drinker, was statistically significantly associated. Additionally, they report a linear dose–response relationship consumption‐LLA
	Low to moderate	OR 1.09 (95% CI 0.91–1.31)	
	High‐level drinkers	OR 1.65 (95% CI 1.28–2.12)	
	0–1 cups per day	OR 1.00 (95% CI 0.89–1.14)	
	1 cups per day	OR 1.04 (95% CI 0.86–1.25)	
	1–2 cups per day	OR 1.10 (95% CI 0.91–1.34)	
	2–3 cups per day	OR 1.30 (95% CI 1.05–1.60)	
	3 cups per day	OR 1.41 (95% CI 1.11–1.78)	
	4–5 cups per day	OR 1.74 (95% CI 1.29–2.35)	
	6 cups per day	OR 2.08 (95% CI 1.46–2.94)	
	10 cups per day	OR 3.02 (95% CI 1.17–7.78)	
	Maternal coffee consumption OR 1.71 (95% CI 1.14–2.55)		
Thomopoulos et al. [34]	Ever vs. never	OR 1.10 (95% CI 0.99–1.22)	High maternal coffee consumption was positively associated with acute lymphoblastic leukemia
	Low to moderate vs. never/lowest	OR 1.01 (95% CI 0.90–1.13)	
	High vs. never/lowest	OR 1.43 (95% CI 1.22–1.68)	
Yan et al. [35]	OR 1.15 (95% CI 0.99–1.32)		No significant association
Blanco‐Lopez et al. [40]	> 2 cups per day vs. no consumption	OR 1.27 (95% CI 1.09–1.43)	Intakes higher that two cups of coffee per day had an increased risk of ALL
	Highest vs. lowest consumption	OR 1.45 (95% CI 1.12–1.89)	

#### 3.4.4 Fruits and Vegetables Consumption

The three SRs addressing this type of maternal exposure [36, 37, 40] seem to suggest that fruit and vegetable consumption during pregnancy is a protective factor against childhood ALL (see Table 6). A total of 10 primary studies were included in these reviews, with an overlap frequency of 40% (four studies repeated across SRs). Due to its quality and recent publication time, the study by Blanco et al. [40] was considered the best SR in this subgroup analysis. Said review reports that there is not a significant association between vegetable consumption during pregnancy and childhood ALL risk, but that fruit consumption behaved as a protective factor [40], which is also informed in the other two reviews [36, 37]. Regarding vegetable consumption during pregnancy, Dessypris et al. report findings in favor of its protective role against childhood ALL development (OR 0.51; 95% CI 0.28–0.94) [37]. Additionally, Blanco et al. and Abiri et al. highlight that carotenoid‐rich foods showed better benefits in terms of this protective role [36, 40].

**TABLE 6:** Reported associations between fruit and vegetable intake during pregnancy and the development of childhood ALL.

References	Measures		Main results
Abiri et al. [36]	Significant inverse association in three studies, but two reported a marginally significant relationship^a^		May reduce risk
	Carrots, beans, and green beans may reduce risk		
	Carotenoids could be probably potent protective compounds		
Dessypris et al. [37]	Fruits	OR, 0.81; 95% CI (0.67–0.99)	Significant inverse association (fruits, vegetables, and legumes)
	Vegetables	OR, 0.51; 95% CI (0.28–0.94)	
	Legumes	OR, 0.76; 95% CI (0.62–0.94)	
Blanco‐Lopez et al. [40]	Fruits	OR, 0.71; 95% CI (0.59–0.86)	Fruits (> 2 servings daily vs. less) was inversely associated
	Vegetables	OR, 0.88; 95% CI (0.69–1.11)	

^a^ This systematic review reports the measures of association that are informed by the primary studies; it does not provide a single (pooled) measure of association.

#### 3.4.5 Folic Acid Supplementation

Folic acid supplementation was addressed by four SRs that included a total of 27 primary studies, of which 11 were repeated across SRs for a 40.7% overlap. Three SRs reported a suggestive association of folic acid supplementation during pregnancy as a protective factor against the development of childhood ALL (see Table 7). Ismail et al. and Blanco‐Lopez et al. found similar ORs (OR 0.75; 95% CI 0.66–0.86 and OR 0.77; 95% CI 0.59–1.01, respectively) [39, 40]; however, due to the latter confidence interval, establishing an association is not possible. On the other hand, Abiri et al., based on a study that groups the results according to the daily micrograms of folic acid ingested during pregnancy, conclude that this protective association is significant regardless of the ingested amount [36].

**TABLE 7:** Reported associations between folic acid intake during pregnancy and the development of childhood ALL.

References	Measures		Main results
Abiri et al. [36]	Only one study about dietary folate (mcg)^a^ 395–454: OR 0.68 (95% Cl 0.44–1.06) 454–524: OR 0.58 (95% Cl 0.37–0.91) 524‐624: OR 0.44 (95% Cl 0.27–0.71) 624: OR 0.70 (95% Cl 0.44–1.12)		Folate as a cytoprotective
Dessypris et al. [37]	Before pregnancy	OR 0.69 (95% CI 0.50–0.95)	Associated with decreased risk
	During pregnancy	OR 0.87 (95% CI 0.57–1.34)	
Ismail et al. [39]	OR 0.75 (95% CI 0.66–0.86)		Folic acid supplementation: associated with protective effect
Blanco‐Lopez et al. [40]	OR 0.77 (95% CI 0.59–1.01)		Modest inverse association

^a^ This systematic review reports the measures of association that are informed by the primary studies; it does not provide a single (pooled) measure of association.

## 4 Discussion

An advanced search for SRs was performed, followed by the extraction of their results and quality assessment using AMSTAR II [26]. Two of the 13 SRs included were not considered for analysis purposes since they were graded as being critically low‐quality reviews. The classification of the remaining studies according to the AMSTAR II assessment tool ranged from low to high‐quality reviews. Furthermore, the frequency of overlap of primary studies ranged from 20% to 50%, which clearly decreases the certainty of the findings to be discussed below.

In the case of alcohol consumption during pregnancy, we did not find SRs supporting its association with the risk of childhood ALL. However, during the data extraction phase, we found multiple studies reporting that alcohol consumption during pregnancy is associated with acute myeloid leukemia risk [30, 38]. Therefore, an in‐depth analysis of the pathophysiological mechanisms underlying this finding is necessary, and future studies on this topic should organize their data according to the types or degrees of alcohol in order to analyze the influence of variables that were not considered. Likewise, we recommend reconsidering the data collection methods, as surveys and telephone interviews do not guarantee reliable results and may introduce potential information bias. Undoubtedly, other factors that can affect the measurement of these variables should be considered, including recall issues regarding intake, quantities, or frequency (more closely related to recall bias, exposure misclassification, or underreporting), as well as social bias, among others.

Considering the other types of leukemia and other diseases such as fetal alcohol spectrum disorders [41], that have been associated with alcohol consumption during pregnancy, in addition to the poor evidence on an intake amount that conditions the occurrence of adverse outcomes in the offspring, alcohol consumption during pregnancy is not recommended despite the findings reported in this study.

It is therefore necessary to consider biological mechanisms or the action of bioactive components in coffee that may either reduce or increase cancer risk, as this beverage contains more than 100 compounds, among which cafestol and kahweol have demonstrated anticancer and anti‐inflammatory properties [42]. Phenolic compounds—specifically chlorogenic acids—have been documented to possess antioxidant properties and to mediate oxidative stress; however, they have also been associated with potentially carcinogenic effects due to DNA damage. The latter has been widely discussed, particularly in relation to the inhibition of topoisomerase II, an enzyme that modifies DNA topology to facilitate replication, transcription, and recombination. The concern here is that inhibition may lead to DNA strand breaks, which could promote aberrant recombination, illegitimate chromosomal translocations, and the formation of chimeric proteins that may disrupt hematopoiesis [42, 43].

However, we cannot conclude with certainty that coffee per se increases the risk of childhood ALL, nor can we rule out the existence of a related confounding variable. Furthermore, recent studies contradict these findings by reporting an association between high coffee intake and lower cardiovascular risk and a decreased incidence of hepatic and endometrial prostate cancer, among others [44, 45, 46].

For the time being, we adhere to the maximum recommended coffee intake during pregnancy, that is, 200 mg of caffeine, as established by the National Health Service (NHS) [47] and the European Food Safety Association (EFSA) [44] in relation to other complications and adverse effects unrelated to ALL that have been associated with coffee consumption.

On the other hand, it is not possible to make a general statement about the impact of animal protein consumption during pregnancy on the risk of childhood ALL. The only significant association of its consumption during pregnancy as a protective factor would seem to come from fish intake; however, the evidence supporting this association is not sufficient, provided that this conclusion derives from the same primary studies, as all of them were included in the three SRs that addressed this type of maternal exposure. In addition, despite there being many studies reporting an association between the intake of processed meats and an increased risk of gastric, colorectal, and breast cancer [48], we did not find any evidence suggesting an association with the development of childhood ALL. Nevertheless, it is worth noting that our findings do not contradict the universal recommendation of having a balanced diet rich in macronutrients during pregnancy [49].

As with fish consumption, the results found could favor the consumption of fruits and vegetables during pregnancy, especially those rich in beta‐carotenes. However, there is insufficient information on the amount, type of preparation, or frequency of consumption necessary to obtain such benefits. For the time being, it is not possible to issue a special fruit and vegetable intake recommendation during pregnancy other than that made by the World Health Organization (WHO) for the general population [49]. Furthermore, the conclusions resulting from the associations of fruits and vegetables and fish consumption during pregnancy as protective factors against the development of childhood ALL need to be analyzed, considering that, of the exposures considered in the present review, these were the ones with the highest frequency of overlap of primary studies (40% and 50%, respectively).

Finally, a protective association between folic acid supplementation during pregnancy and the risk of childhood ALL is proposed. This would represent an additional benefit derived from the mandatory folic acid supplementation during pregnancy policy and the fortification of food with folic acid policy established in some countries as a strategy to prevent neural tube defects [50]. However, the certainty of this conclusion is low due to an overlap of primary studies of 40.7% across SRs.

There is still not a consensus on this topic. For example, the European Union opposes a mandatory fortification policy due to the unknown effects of high doses of folic acid in the long term in the general population and taking into account that women of childbearing age are the main target population of this strategy [51]. Currently, there are controversial studies reporting an association between folic acid intake and an increased risk of colorectal and prostate cancer [50, 51], while others support the policy by reporting a protective role against cardiovascular and neurodegenerative diseases [52, 53].

Considering the existing controversy and the need for further research to conclude with certainty its additional benefits, recommending a model of folic acid supplementation during pregnancy other than those established in each country is not possible.

### 4.1 Strengths and Limitations

One of the strengths of this overview includes its methodology, since multiple databases in different languages were searched, which made it possible to cover more information; besides, the AMSTAR II tool was used for assessing the quality of the SRs selected for analysis [26]. In addition, the underlying distribution of the results by exposure subgroups allowed for a better organization and a differential approach in their analysis, since variables, hypotheses, and individual characteristics of each subgroup were considered. Finally, the focus given to ALL, a hematological neoplasm that is usually studied together with other types of leukemia without considering its own etiological, epidemiological, and pathophysiological differences, stands out.

With respect to its limitations, we highlight the difficulty of putting theoretical results into practice, as well as the dependence on the quality of the selected reviews and the primary studies they include. Also, we recognize that it is possible that not all existing studies on the subject were included in the present review, but we are certain that the probability of this happening was reduced by the systematic methodology used in our study. On the other hand, due to the variability in the results reported by the SRs, it was not possible to draw conclusions in some cases, nor make a meta‐analysis, so we propose that future studies on this topic should standardize the reporting of their results. Finally, an important element to be considered is the overlapping of primary studies across SRs, particularly fish consumption, since the SRs by Abiri et al. and Blanco‐Lopez et al. base their claims on the association of fish consumption as a protective factor against childhood ALL on the same two primary studies [36, 40]. To deal with this problem, we presented the data overlap matrices for each exposure type and, where possible, we gave greater weight to the best SR in each exposure subgroup based on the following parameters: quality, number of studies included, and year of publication.

### 4.2 Biases

Primary studies with evaluated SRs are predisposed to selection bias because they are retrospective, even more if the information about maternal diet was obtained through surveys, since there is the possibility that the mothers of children and adolescents with ALL gave more value to the evaluated exposures to justify the development of the disease. Likewise, these questionnaires can result in information bias, as answers depend on the participants' memory, which makes it difficult to predict the impact of an error, because the real association can be underestimated or overestimated. In addition, since these are observational studies, ensuring that the population and exposure studied are consistent is difficult, thus increasing the confounding factor. Finally, it is worth mentioning the bias derived from funding, which was reported in five of the 13 SRs included, and the fact that data presented by three of these SRs were part of the results reported in our overview. However, we consider the risk of bias low as the best reviews were selected using the AMSTAR II tool [26].

## 5 Conclusion

In this review, we considered the available evidence on the association between maternal diet during pregnancy and the risk of childhood ALL, focusing the analysis on five specific dietary factors. The consumption of fruit, vegetables, animal protein (fish) and folic acid during pregnancy appeared to be a protective factor against the development of this type of cancer, but the certainty of this statement is low due to the high overlap of studies in different SR. In order to provide concrete answers, a different study design is needed, one that addresses shortcomings in the quality of the studies and at the same time allows gaps in the literature to be filled. Overview studies such as this one provide an ideal starting point, as they synthesize the existing information, detail the presentation and quality of the studies, and review the scientific evidence as comprehensively as possible. As far as alcohol is concerned, no clear association has been found with ALL; however, it does have a detrimental effect on other diseases. The design of primary studies on the role of alcohol and hematological neoplasms is important in view of the preventive role it could play during the prenatal period. It would also allow a pathophysiological correlation to be established between alcohol consumption and other types of cancer, including the leukemia that are common in adulthood. Regarding coffee, there was a positive association as a risk factor for ALL, directly related to the level of consumption. Future studies on this topic should standardize the reporting of their results, since the high heterogeneity found between studies made it impossible to present a quantitative synthesis of the evidence.

## Author Contributions

**Kathya Torres‐Duarte, Laura Marcela Chávez Rodríguez,** and **Catalina Mora‐Becerra:** conceptualization (equal), data curation (equal), formal analysis (equal), writing – original draft (equal), and writing – review and editing (equal). **Jaime Moreno‐Chaparro:** methodology (equal), data curation (equal), formal analysis (equal), writing – original draft (equal), and writing – review and editing (equal). **Hernando Gaitán‐Duarte:** methodology (equal), data curation (equal), formal analysis (equal), and writing – review and editing (equal).

## Ethics Statement

The authors have nothing to report.

## Consent

The authors have nothing to report.

## Conflicts of Interest

The authors declare no conflicts of interest.

## Supporting information


Data S1.

Data S2.

Data S3.

Data S4.

## Data Availability

The data that support the findings of this study are available from the corresponding author upon reasonable request.
