# Supplementary material for: Association Between Maternal Diet During Pregnancy and the Risk of Childhood Acute Lymphoblastic Leukemia. An Overview
Source: Cancer Rep (Hoboken). 2025 Jun 11;8(6):e70231. doi: 10.1002/cnr2.70231 (PMC12152502; doi:10.1002/cnr2.70231)
Supplement: Supplementary file 1 — Data S1. [file CNR2-8-e70231-s002.docx]

**Annex 1.** Search algorithms and results by database.

| **Search Report #1** | | |
| --- | --- | --- |
| **Database** | MEDLINE | |
| **Platform** | Pubmed | |
| **Date range** | 1966 - 2023 | |
| **Language restrictions** | English, Spanish, French and Portuguese | |
| **Date search** | 03/10/2023 | |
| **Search** | 1. leukemia[MeSH Terms] (254,677)  2. Leukemia[Title/Abstract] AND (acute[Title/Abstract] OR lymphoblastic[Title/Abstract] OR lymphoid[Title/Abstract] OR lymphoma[Title/Abstract] OR hematologic[Title/Abstract] OR hematopoietic[Title/Abstract] OR haematologica[Title/Abstract] OR haematopoietic[Title/Abstract] OR blood[Title/Abstract]) (154,555)  3. #1 OR #2 (300,315)  4. Mother[Title/Abstract] OR pregnan*[Title/Abstract] OR maternal[Title/Abstract] OR parent*[Title/Abstract] OR infant*[Title/Abstract] OR infancy[Title/Abstract] OR children*[Title/Abstract] OR childhood[Title/Abstract] OR breastfeeding[Title/Abstract] OR "breast feeding"[Title/Abstract] OR lactation[Title/Abstract] OR lactating[Title/Abstract] (2,809,346)  5. Nutrition*[Title/Abstract] OR nutrient*[Title/Abstract] OR nutrit*[Title/Abstract] OR diet*[Title/Abstract] OR eat*[Title/Abstract] OR food[Title/Abstract] OR breastfeeding[Title/Abstract] OR "breast feeding"[Title/Abstract] OR lactation[Title/Abstract] OR supplement*[Title/Abstract] OR Fruit*[Title/Abstract] OR Vegetable*[Title/Abstract] OR meat*[Title/Abstract] OR folate*[Title/Abstract] OR folic*[Title/Abstract] OR "Dietary Supplements"[Title/Abstract] OR "coffee"[Title/Abstract] OR "caffeine"[Title/Abstract] OR alcohol[Title/Abstract] OR Alcoholic[Title/Abstract] OR drink*[Title/Abstract] OR beer*[Title/Abstract] OR wine*[Title/Abstract] OR beverage*[Title/Abstract] (#2,76,391)  6. #4 AND #5 (374.501)  7. #3 AND #6 (1,724)  8. Review*[Title/Abstract] OR (Review[Title/Abstract] AND (Systematic[Title/Abstract] OR scoping[Title/Abstract] OR narrative[Title/Abstract] OR metaanalisis[Title/Abstract] OR meta-analysis[Title/Abstract] OR metaanalysis[Title/Abstract])) (2,801,174)  9. #7 AND #8 (239) | |
| **Results** | **239** | |

| **Search Report #2** | | |
| --- | --- | --- |
| **Database** | EMBASE | |
| **Platform** | EMBASE | |
| **Date range** | 1947 - 2023 | |
| **Language restrictions** | Spanish, Portuguese, French and English | |
| **Date search** | 03/10/2023 | |
| **Search** | 1. 'leukemia'/exp (438,049)  2. (leukemia:ab,ti OR leucocyth:ab,ti) AND (acute:ab,ti OR lymphoblastic:ab,ti OR lymphoid:ab,ti OR lymphoma:ab,ti OR hematologic:ab,ti OR hematopoietic:ab,ti OR haematologic:ab,ti OR haematopoietic:ab,ti OR blood:ab,ti) (234,654)  3. #1 OR #2 (471,184)  4. Mother:ab,ti OR 'pregnancy'/exp OR pregnan*:ab,ti OR maternal:ab,ti OR parent*:ab,ti OR infant*:ab,ti OR infancy:ab,ti OR 'Child'/Exp OR children*:ab,ti OR childhood:ab,ti OR breastfeeding:ab,ti OR "breast feeding":ab,ti OR lactation:ab,ti OR lactating:ab,ti (5,302,450)  5. Nutrition*:ab,ti OR nutrient*:ab,ti OR nutrit*:ab,ti OR diet*:ab,ti OR eat*:ab,ti OR food:ab,ti OR breastfeeding:ab,ti OR "breast feeding":ab,ti OR lactation:ab,ti OR supplement*:ab,ti OR Fruit*:ab,ti OR Vegetable*:ab,ti OR meat*:ab,ti OR folate*:ab,ti OR folic*:ab,ti OR "Dietary Supplements":ab,ti OR "coffee":ab,ti OR "caffeine":ab,ti OR alcohol:ab,ti OR Alcoholic:ab,ti OR drink*:ab,ti OR beer*:ab,ti OR wine*:ab,ti OR beverage*:ab,ti (3,133,600)  6. #4 AND #5 (577,265)  7. #3 AND #6 (4,279)  8. 'Review'/exp OR Review*:ab,ti OR (Review:ab,ti AND (Systematic:ab,ti OR scoping:ab,ti OR narrative:ab,ti OR metaanalisis:ab,ti OR meta-analysis:ab,ti OR metaanalysis:ab,ti)) (5,272,533)  9. #7 AND #8 (844) | |
| **Results** | **844** | |

| **Search Report #3** | | |
| --- | --- | --- |
| **Database** | SCOPUS | |
| **Platform** | SCOPUS | |
| **Date range** | 2004 - 2023 | |
| **Language restrictions** | Spanish, Portuguese, French and English | |
| **Date search** | 03/10/2023 | |
| **Search** | 1. TITLE-ABS ( leukemia OR leucocyth AND ( acute OR lymphoblastic OR lymphoid OR lymphoma OR hematologic OR hematopoietic OR haematologic OR haematopoietic OR blood ) ) (199,766)  2. TITLE-ABS ( mother OR pregnan* OR maternal OR parent* OR infant* OR infancy OR children* OR childhood OR breastfeeding OR "breast feeding" OR lactation OR lactating ) (4,020,415)  3. TITLE-ABS ( nutrition* OR nutrient* OR nutrit* OR diet* OR eat* OR food OR breastfeeding OR "breast feeding" OR lactation OR supplement* OR fruit* OR vegetable* OR meat* OR folate* OR folic* OR "Dietary Supplements" OR "coffee" OR "caffeine" OR alcohol OR alcoholic OR drink* OR beer* OR wine* OR beverage* ) (4,972,897)  4. #2 AND #3 (518,748)  5. #1 AND #4 (1,706)  6. TITLE-ABS ( review* OR ( review AND ( systematic OR scoping OR narrative OR metaanalisis OR meta-analysis OR metaanalysis ) ) ) (4,907,863)  7. #5 AND #6 (261) | |
| **Results** | **261** | |

| **Search Report #4** | | |
| --- | --- | --- |
| **Database** | LILACS | |
| **Platform** | LILACS | |
| **Date range** | 1985 - 2023 | |
| **Language restrictions** | Spanish, Portuguese, French and English | |
| **Date search** | 03/10/2023 | |
| **Search** | (leukemia) AND ((mother OR pregnan* OR maternal OR infant* OR child*) AND (nutrition* OR diet* OR eat* OR food OR supplement* OR fruit* OR vegetable* OR meat* OR folic* OR coffee OR alcohol)) AND ( db:("LILACS") AND type_of_study:("systematic_reviews")) (4) | |
| **Results** | **4** | |

| **Search Report #5** | | |
| --- | --- | --- |
| **Database** | Web of Science | |
| **Platform** | Web of Science (Core collection) | |
| **Date range** | 2000 - 2023 | |
| **Language restrictions** | Spanish, Portuguese, French and English | |
| **Date search** | 03/10/2023 | |
| **Search** | 1. TS=(Leukemia AND (acute OR lymphoblastic OR lymphoid OR lymphoma OR hematologic OR hematopoietic OR haematologica OR haematopoietic OR blood)) (424)  2. TS=(Mother OR pregnan* OR maternal OR parent* OR infant* OR infancy OR children* OR childhood OR breastfeeding OR "breast feeding" OR lactation OR lactating) (#2,899,218)  3. TS=(Nutrition* OR nutrient* OR nutrit* OR diet* OR eat* OR food OR breastfeeding OR "breast feeding" OR lactation OR supplement* OR Fruit* OR Vegetable* OR meat* OR folate* OR folic* OR "Dietary Supplements" OR "coffee" OR "caffeine" OR alcohol OR Alcoholic OR drink* OR beer* OR wine* OR beverage*) (3,452,598)  4. #2 AND #3 (425,508)  5. #4 AND #1 (2,138)  6. TS=(Review* OR (Review AND (Systematic OR scoping OR narrative OR metaanalisis OR meta-analysis OR metaanalysis))) (2,899,218)  7. #5 AND #6 (424) | |
| **Results** | **424** | |
