# Supplementary material for: Association Between Maternal Diet During Pregnancy and the Risk of Childhood Acute Lymphoblastic Leukemia. An Overview
Source: Cancer Rep (Hoboken). 2025 Jun 11;8(6):e70231. doi: 10.1002/cnr2.70231 (PMC12152502; doi:10.1002/cnr2.70231)
Supplement: Supplementary file 2 — Data S2. [file CNR2-8-e70231-s001.pdf]

Annex 2. Overlap of primary studies in included systematic reviews

2.1 Alcohol group

	Van Steensel-	Severson et	van Duijn et	Ross et al,	Shu et al,	Petridou et al,	Schuz et al,	Wen et al,	Alexander et	Costas et al,	Infante Rivard	Mejia-	Clavel et al,	Menegaux et	Kabuto et al,	MacArthur et	Monge et	Rudant, 2008	Liu et al, 2009	Canfield et
**Latino-Martel, et Al., 2010**	x	x	x	x	x	x	x	x	x	x	x	x	x	x	x	x	x	x	x	
**Zhang, et Al, 2010**								x						x		x		x		x
**Brisson, et Al., 2015**																				
**Yan, et Al., 2015**					x	x	x	x			x			x				x		
**Behnaz Abiri, et Al., 2016**																				
**Karalexi, et Al., 2017**	x				x	x		x	x		x			x	x	x	x	x		

**Quality**		Krajinovic	Chen et al.,	Barnette et	Klotz et al.,	Urayama et	Beuten et	Swinney et	Chokkaling	Ferreira et	Bonaventur	Silveira et	Zanrosso et	De Aguilar	Slater et	Milne et al.,	Metayer et	Castro-	Abdi-korek	Orsi et al.,	Spreche et
Moderate	**Latino-Martel, et Al., 2010**																				
Low	**Zhang, et Al, 2010**																				
Low	**Brisson, et Al., 2015**	x	x	x	x	x	x	x	x			x	x	x							
High	**Yan, et Al., 2015**					x					x					x		x			x
Low	**Behnaz Abiri, et Al., 2016**									x	x				x	x				x	
High	**Karalexi, et Al., 2017**		x							x					x	x	x	x	x	x	x

**Quality**		Rangel et	Wunsch-	Canalle et	Yamaguti	Linet et al.,	Hatch et	Freedman	Hjalgrim et	Kwan et	Ronsenba	Chang et	Hughes et	Simpson et	Blair et al,	Bartley et	Feller et	Bailey et	Reid et al.,	Oksuzyan	Larfors et
Moderate	**Latino-Martel, et Al., 2010**																				
Low	**Zhang, et Al, 2010**																				
Low	**Brisson, et Al., 2015**			x	x																
High	**Yan, et Al., 2015**					x	x	x	x	x	x	x	x	x	x	x	x	x	x	x	x
Low	**Behnaz Abiri, et Al., 2016**																				
High	**Karalexi, et Al., 2017**	x	x																		

Quality		Ding et al.,	Roman et	Diamantar	Lariou et	Farioli et	Schraw et	Lautner-	Borst et	Ulusoy et	Tumer et	Joseph et	Hattori et	Pakakasa	Gatedee	Pongstap	Rimando	Wang et	Yeoh et	Chan et	Razmkha
Moderate	**Latino-Martel, et Al., 2010**																				
Low	**Zhang, et Al, 2010**																				
Low	**Brisson, et Al., 2015**							x	x	x	x	x	x	x	x	x	x	x	x	x	x
High	**Yan, et Al., 2015**	x	x	x	x	x	x														
Low	**Behnaz Abiri, et Al., 2016**																				
High	**Karalexi, et Al., 2017**																				

Quality		Wiemels	Alves et	Jamrozia	Lanciotti	Pigullo et	Semsei et
Moderate	**Latino-Martel, et Al., 2010**						
Low	**Zhang, et Al, 2010**						
Low	**Brisson, et Al., 2015**	x	x	x	x	x	x
High	**Yan, et Al., 2015**						
Low	**Behnaz Abiri, et Al., 2016**						
High	**Karalexi, et Al., 2017**						

2.2 Animal protein group

QUALITY		Ross et al., 1996	Jensen et al.,	Spector et al.,	Petridou et al.,	Kwan et al., 2009	Bailey et al., 2012	Peters et al., 1997	Sarasua et al.,	Ajrouche et al.,	Singer et al., 2016
Low	**Abiri et al, 2016**	x	x		x	x	x	x			
Low	**Dessypris et al, 2017**	x	x	x	x	x			x	x	
High	**Blanco-Lopez et al., 2023**		x		x	x			x		x

2.3 Coffee

Quality		Ross et al., 1996	Petridou et al., 1997	Petridou et al., 2005	Clavel et al., 2004	Menegaux et al., 2005	Menegaux et al., 2007	Milne et al., 2011	Bonaventure et al.,	Peters et al., 1994	Orsi et al., 2015	Linet et al., 1997	Hatch et al., 1998	Schuz et al., 1999	Shu et al., 1999	Infante-Rivard et al.,	Wen et al., 2002	Freedman et al., 2001	Hjalgrim et al., 2004	Kwan et al., 2004	Jourdan-Da-Silva et al.,
Low	**Cheng et al., 2013**	x	x		x	x	x	x	x												
Moderate	**Thomopoulus et al., 2015**	x	x	x	x	x	x	x	x	x	x										
High	**Yan et al., 2015**		x	x		x	x	x	x			x	x	x	x	x	x	x	x	x	x
High	**Blanco-Lopez et al., 2023**	x				x	x	x	x		x										

Quality		Rosenbaum et al., 2005	Chang et al., 2006	Hughes et al., 2007	Simpson et al., 2007	Blair et al., 2008	Bailey et al., 2010	Bartley et al., 2010	Reid et al., 2011	Castro-Jimenez et al.,	Urayama et al., 2011	Oksuzyan et al., 2012	Bonaventure et al., 2012	Larfors et al., 2012	Ding et al., 2012	Roman et al., 2013	Rudant et al., 2013	Diamantaras et al., 2013	Lariou et al, 2013	Farioli et al., 2014	Schraw et al., 2014
Low	**Cheng et al., 2013**																				
Moderate	**Thomopoulus et al., 2015**																				
High	**Yan et al., 2015**	x	x	x	x	x	x	x	x	x	x	x	x	x	x	x	x	x	x	x	x
High	**Blanco-Lopez et al., 2023**																				

2.4 Fruits and legumes group

**Quality**	Systematic Review	Ross et al.,	Jensen et	Spector et	Petridou et	Kwan et al.,	Bailey et al.,	Peters et	Sarasua et	Ajrouche et	Singer et
Low	**Behnaz Abiri, et Al., 2016**	x	x	x	x	x	x	x			
Low	**Dessypris et al, 2017**	x	x		x	x			x	x	
High	**Blanco-Lopez et al., 2023**		x		x	x			x		x

2.5 Folic acid group

**Quality**	Systematic Review	Ross et	Ross et	Jensen et	Spector	Petridou	Kwan et	Bailey et	Bailey,	Milne et	Milne et	Linabery	Mc	Metayer	Dockerty
Low	**Behnaz Abiri, et Al., 2016**	x		x	x	x	x	x							
Low	**Dessypris et al, 2017**									x		x	x	x	x
Moderate	**Ismail et al., 2019**		x					x	x	x	x	x			
High	**Blanco-Lopez et al., 2023**			x			x	x		x				x	x

**Quality**	Systematic Review	Amitary et	Ajrouche	Preston-	Wen et al.,	Shaw et	Schuz et	Johnson	Stalberg et	Ortega-	Amigou et	Thompson	Singer et	Bonaventu
Low	**Behnaz Abiri, et Al., 2016**													
Low	**Dessypris et al, 2017**	x	x											
Moderate	**Ismail et al., 2019**	x	x	x	x	x	x	x	x	x	x		x	
High	**Blanco-Lopez et al., 2023**		x								x	x	x	x
