# Supplementary material for: Association Between Maternal Diet During Pregnancy and the Risk of Childhood Acute Lymphoblastic Leukemia. An Overview
Source: Cancer Rep (Hoboken). 2025 Jun 11;8(6):e70231. doi: 10.1002/cnr2.70231 (PMC12152502; doi:10.1002/cnr2.70231)
Supplement: Supplementary file 3 — Data S3. [file CNR2-8-e70231-s004.docx]

**Annex 3.** Reasons for rejection of selected articles.

|  | **Reference** | **Title** | **Reason for rejection** |
| --- | --- | --- | --- |
| **1** | Schuster-Kolbe, J., et Al. 1994 | Smoking and cancer risk | Not an exposure group. |
| **2** | Shu, X., 1997 | Epidemiology of childhood leukemia | Not a systematic review |
| **3** | Craft, A.W., 1998. | Childhood cancer: improved prospects for survival but is prevention possible? | Not a systematic review |
| **4** | Lightfoot, t., et Al., 2004 | Causes of childhood leukaemia and lymphoma | Not a systematic review |
| **5** | Bunin, G. R., 2004. | Nongenetic causes of childhood cancers: evidence from international variation, time trends, and risk factor studies | Not a systematic review |
| **6** | Mejía Aranguré, et Al., 2005 | Acute leukemias epidemiology in children. Part 1 | Not a systematic review |
| **7** | Buffler, et Al., 2005 | Environmental and genetic risk factors for childhood leukemia: Appraising the evidence | Not a systematic review |
| **8** | Mcnally, R. et Al., 2006. | Environmental factors and childhood acute leukemias and lymphomas | Not a systematic review |
| **9** | Kim, Y. 2007 | Folic acid fortification and supplementation - Good for some but not so good for others | Not a systematic review |
| **10** | Belson M, et Al., 2007 | Risk factors for acute leukemia in children: A review | Not a systematic review |
| **11** | Goh, et Al., 2007 | Prenatal multivitamin supplementation and rates of pediatric cancers: A meta-analysis | Not an exposure group. |
| **12** | Tower RL, et Al., 2007. | Diet as a potential moderator for genome stability and immune response in pediatric leukemia | Insufficient information |
| **13** | [Papandreou, D.](https://www.emerald.com/insight/search?q=D.%20Papandreou) et Al., 2008. | Are maternal diet, birth weight, breastfeeding and antioxidants playing a role in pediatric leukemia? A review | Not a systematic review |
| **14** | Goh, et Al., 2008 | Prenatal supplementation with multivitamins and the incidence of pediatric cancers: Clinical and methodological considerations | Not a systematic review |
| **15** | Infante-Rivard, 2008 | Chemical risk factors and childhood leukaemia: a review of recent studies dagger | Not an exposure group. |
| **16** | Burdge GC, et Al., 2009. | Nutrition in early life, and risk of cancer and metabolic disease: alternative endings in an epigenetic tale? | Not a systematic review |
| **17** | Milne, et Al., 2010 | Maternal folate and other vitamin supplementation during pregnancy and risk of acute lymphoblastic leukemia in the offspring | Not a systematic review |
| **18** | Eden, T. 2010 | Aetiology of childhood leukaemia | Not a systematic review |
| **19** | Arab., 2010. | Epidemiologic evidence on coffee and cancer | Not a systematic review |
| **20** | Andres, S., et Al., 2011 | Risks and benefits of dietary isoflavones for cancer | Not a systematic review |
| **21** | Shen, X., et Al., 2013 | Maternal flavonoids intake and infant leukemia | Language not supported |
| **22** | Kaur, P., et Al., 2013 | The epigenome as a potential mediator of cancer and disease prevention in prenatal development | Not a systematic review |
| **23** | Pistollato et Al., 2015. | Plant-Based and Plant-Rich Diet Patterns during Gestation: Beneficial Effects and Possible Shortcomings | Not a systematic review |
| **24** | Jin, et Al., 2016 | A review of risk factors for childhood leukemia | Not a systematic review |
| **25** | Whitehead, et Al., 2016 | Childhood Leukemia and Primary Prevention | Not a systematic review |
| **26** | Alicandro, et Al., 2017 | Coffee and cancer risk: A summary overview | Not a systematic review |
| **27** | Cantarella., 2017. | Folate deficiency as predisposing factor for childhood leukaemia: A review of the literature | Not a systematic review |
| **28** | Dong, et Al., 2019. | Tobacco smoke exposure and the risk of childhood acute lymphoblastic leukemia and acute myeloid leukemia A meta-analysis | Not an exposure group. |
| **29** | Cao, et Al., 2020 | Paternal Smoking Before Conception and During Pregnancy Is Associated With an Increased Risk of Childhood Acute Lymphoblastic Leukemia: A Systematic Review and Meta-Analysis of 17 Case-Control Studies | Not an exposure group. |
| **30** | Chrysoula., 2020. | The association between maternal folic acid supplementation and risk of childhood leukeamia: a systematic review focusing on the potential role of epigenetic mechanisms | Insufficient information |
| **31** | Baptiste-Roberts, et Al., 2020 | Caffeine exposure during pregnancy: Is it safe? | Not a systematic review |
| **32** | James, 2021. | Maternal caffeine consumption and pregnancy outcomes: A narrative review with implications for advice to mothers and mothers-to-be | Not a systematic review |
| **33** | Wang, et Al., 2021. | Diet as a potential moderator for genome stability and immune response in pediatric leukemia | Not a systematic review |
| **34** | Onyije, et Al., 2022 | Environmental Risk Factors for Childhood Acute Lymphoblastic Leukemia: An Umbrella Review | Not a systematic review |
| **35** | Huybrechts, 2023. | The role of nutrition and other lifestyle factors in paediatric oncology | Not a systematic review |
